# Endoplasmic reticulum stress-mediated inflammatory signaling pathways within the osteolytic periosteum and interface membrane in particle-induced osteolysis

**DOI:** 10.1007/s00441-015-2205-9

**Published:** 2015-05-26

**Authors:** Guoyin Liu, Naicheng Liu, Yuansheng Xu, Yunfan Ti, Jiangning Chen, Jianmin Chen, Junfeng Zhang, Jianning Zhao

**Affiliations:** Department of Orthopaedics, Jinling Hospital affiliated to the School of Medicine, Nanjing University, Nanjing, 210093 China; State Key Laboratory of Pharmaceutical Biotechnology, School of Life Sciences, Nanjing University, Nanjing, 210093 China; Department of Orthopaedics, Jingdu Hospital, Nanjing, 210002 China

**Keywords:** Periprosthetic inflammatory osteolysis, Endoplasmic reticulum stress, Aseptic loosening, Wear particles, Inflammatory signaling pathways, Osteoclastogenesis, Human, Mouse

## Abstract

Aseptic loosening secondary to periprosthetic inflammatory osteolysis results from the biological response to wear particles and is a leading cause of arthroplasty failure. The origin of this inflammatory response remains unclear. We aim to validate the definite link between endoplasmic reticulum (ER) stress and particle-induced inflammatory signaling pathways in periprosthetic osteolysis. We examine the histopathologic changes of osteolysis and the expression of specific biomarkers for ER-stress-mediated inflammatory signaling pathways (IRE1α, GRP78/Bip, c-Fos, NF-κB, ROS and Ca^2+^). Moreover, pro-inflammatory cytokines (TNF-α, IL-1β and IL-6) and osteoclastogenic molecules (VEGF, OPG, RANKL and M-CSF) were assessed in clinical interface membranes and murine periosteum tissues. We found wear particles to be capable of inducing ER stress in macrophages within clinical osteolytic interface membranes and murine osteolytic periosteum tissues and to be associated with the inflammatory response and osteoclastogenesis. Blocking ER stress with sodium 4-phenylbutyrate (4-PBA) results in a dramatic amelioration of particle-induced osteolysis and a significant reduction of ER-stress intensity. Simultaneously, this ER-stress blocker also lessens inflammatory cell infiltration, diminishes the capability of osteoclastogenesis and reduces the inflammatory response by lowering IRE1α, GRP78/Bip, c-Fos, NF-κB, ROS and Ca^2+^ levels. Thus, ER stress plays an important role in particle-induced inflammatory osteolysis and osteoclastogenic reactions. The pharmacological targeting of ER-stress-mediated inflammatory signaling pathways might be an appealing approach for alleviating or preventing particle-induced osteolysis in at-risk patients.

## Introduction

Total joint replacement by the implantation of a permanently in-dwelling artificial prosthesis is a revolutionary surgical process for enhancing the agility of a patient with joint dysfunction (Klawitter et al. 2011; Rubak et al. 2014; Wooley and Schwarz 2004). However, aseptic loosening secondary to periprosthetic osteolysis, induced by the ensuing adverse biological responses to wear particles, can jeopardize the longevity and prolonged efficacy of the prosthetic components (Beck et al. 2012; Noordin and Masri 2012; Park et al. 2005; Purdue et al. 2006). Wear particles originating at the prosthesis interface can become distributed along the periprosthetic ambience and can occupy neighboring tissues in which they are phagocytosed by tissue-resident cells (Jasty and Smith 1992). These activated cells then secrete an array of pro-inflammatory cytokines (tumor necrosis factor-α [TNF-α], interleukin-1β [IL-1β], and IL-6), osteoclastogenic molecules (vascular endothelial growth factor [VEGF], osteoprotegerin OPG, receptor activator of nuclear kaapa B [RANKL] and macrophage/colony-stimulating factor [M-CSF]) and other mediators of inflammation. This exacerbates the osteolytic responses via multiple biological functions, including the progression of an aggressive interface membrane adjacent to the bone, the differentiation of macrophages into lacunar bone-resorbing osteoclasts and the emancipation of the cytokines mentioned above to attract and enlist additional inflammatory cells that liberate more pro-inflammatory cytokines, osteoclastogenic cytokines and other mediators of inflammation. The cascade perpetuates a positive feedback cycle of inflammation and osteoclastogenesis activation (Burton et al. 2013; Dalal et al. 2012; Ingham and Fisher 2005; Jiang et al. 2013a; Obando-Pereda et al. 2014; Raghunathan et al. 2013; Shimizu et al. 2010; Wang et al. 2010). Eventually, considerable amounts of osteoclast precursor cells and osteoclasts are enlisted and/or activated to absorb the bone, a process that leads to local progressive bone destruction and finally prosthesis loosening.

Accumulating evidence (Goodman et al. 1998; Ingham and Fisher 2005; Jiang et al. 2013b; Kadoya et al. 1998; O’Neill et al. 2013; Obando-Pereda et al. 2014; Shen et al. 2006; Shimizu et al. 2010) has demonstrated that the integral elements underlying bone destruction in periprosthetic osteolysis are macrophages in the osteolytic interface membrane. These cells are mainly involved in inflammation and osteoclastogenesis and have been implicated in the principle pathophysiologic mechanism leading to the development of particle-induced osteolysis. In addition, investigators (Archibeck et al. 2001; Hirashima et al. 2001; Katagiri and Takahashi 2002; Pearle et al. 2007; Teitelbaum 2006) have previously confirmed that macrophages can be recruited to the interface membrane around loosening prostheses by particle-induced continuous inflammation. Moreover, tissue-resident macrophages might be able to differentiate into osteoclasts, which are in charge of osteolysis. Therefore, we considered it probable that macrophages represent both the origin and the impetus for the pathogenic cascade in osteolysis. Thus, the interaction between macrophages and wear particles in the osteolytic interface membrane is probably the factor deciding whether the particle-induced inflammatory response will be dissipated or will develop into irreversible periprosthetic osteolysis.

Over the past few years, many reports have been focused on the various functions of the various cytokines and macrophages (Caruso et al. 2014; Geng et al. 2010b; Jin et al. 2011; Lee et al. 2013; Lin et al. 2014; Mao et al. 2012) and our understanding of the cellular and molecular mechanisms controlling particle-induced inflammatory osteolysis has rapidly improved (Abu-Amer et al. 2007; Caruso et al. 2014; Clohisy et al. 2004; Geng et al. 2010a; Kong et al. 2013; Landgraeber et al. 2009, 2014; Lee et al. 2013; Li et al. 2014; Liu et al. 2009, 2014; Mao et al. 2012; Shetty et al. 2005; Wang et al. 2013; Zhai et al. 2014). However, the way that inflammatory osteolysis originates is not completely understood. A new and attractive underlying mechanism involves the endoplasmic reticulum (ER; Cao and Kaufman 2014; Gorlach et al. 2006; Hotamisligil 2010; Lai et al. 2007; Marciniak and Ron 2006), which is the main cellular site for the folding and trafficking of protein and vital for many molecular functions. As a common pathway of many stress processes, ER is a potentially promising emerging treatment target, partly because its adaptive mechanisms have no time to handle the sustained stresses suffered because of the continuous generation of wear particles in the periprosthetic space. In addition, ER is interconnected with inflammatory signaling pathways by the unfolded protein response and ER stress. Notably, studies assessing the unfolded protein response and ER stress have extended the comprehension of possible mechanisms by which inflammatory signaling pathways can originate (Cao and Kaufman 2014; Gao et al. 2012; Lai et al. 2007; Zhang and Kaufman 2008). The branches of representative ER stress intersect with sundry inflammatory signaling systems, including the activation of the IRE1α/JNK/c-Fos-AP-1 and nuclear factor kappa B (NF-κB) pathways, release of Ca^2+^ from the ER and reactive-oxygen species (ROS) production.

Recently, researches (Christen and Fent 2012; Tsai et al. 2011; Wang et al. 2013; Zhang and Kaufman 2008; Zhang et al. 2012) have shown that the cell fate and growth in the interface membrane, influenced by wear particles, are activated in response to ER stress; in addition, prolonged and enduring ER stress is thought to be essential to the pathogenesis of inflammatory diseases. However, previous work has provided only a limited viewpoint regarding the influence of ER stress on inflammatory osteolysis and reports describing the straight effect of ER stress on particle-induced inflammation and osteoclastogenesis are scarce. Thus, we hypothesized that particle-induced inflammatory signaling pathways within the osteolytic interface membrane in periprosthetic osteolysis are mediated by ER stress and that the inhibition of the ER-stress response in osteolytic tissue macrophages, which can further cause changes in particle-induced inflammation and osteoclastogenesis, is undoubtedly a good therapeutic option. Therefore, in our study, we determined the potential effect of ER stress on wear-particle-induced osteolysis, osteoclastogenesis and macrophage inflammation within osteolytic tissues in an effort to identify the mechanism of the inflammatory response caused by wear particles.

## Materials and methods

### Ethics statement

This study conducted in Jinling Hospital affiliated to the School of Medicine, Nanjing University (China) was approved by the local ethics institutional review board (Jinling Hospital Ethics Committee). Written informed consent was obtained from each participant or from guardians in the case of minors. In addition, all animal studies were approved by the Committee on the Ethics of Animal Experiments of Nanjing University (permit no. 2011–039) and followed “The Institutional Guidelines for the Care and Use of Laboratory Animals at Nanjing University”. The surgical procedure was implemented under sodium pentobarbital anesthesia and all endeavors were undertaken to minimize suffering.

### Reagents

Bovine serum albumin (BSA), 4-phenylbutyric acid (4-PBA), thapsigargin (Tg) and a cocktail of protease inhibitors were all purchased from Sigma-Aldrich (St. Louis, Mo., USA). RIPA lysis buffer was purchased from Beyotimme (Nantong, China). The toluidine blue and hematoxylin and eosin (HE) staining kits were purchased from Jiancheng Biotech (Nanjing, China). Enzyme-linked immunosorbent assay (ELISA) kits for examining mouse/human TNF-α, IL-1β, IL-6 and M-CSF were purchased from 4A Biotech (Beijing, China). Primary antibodies to OPG and RANKL used in immunohistochemistry were purchased from Abcam (USA). Primary antibodies to VEGF (immunohistochemistry) were purchased from Santa Cruz (USA).

### Particle preparation

Nanoparticles of TiAl6V4 (TiNPs) and CoCrMo (CoNPs), provided by Dr. Zhenzhong Zhang (College of Materials Science and Engineering of Nanjing University of Technology), had a mean diameter of 51.7 nm as determined by transmission electron microscopy (TEM). Metal nanoparticles were manipulated in an aseptic milieu. Briefly, particles were washed five times with 70 % ethanol and incubated for 48 h for sterilization at ambient temperature. Then, the metal nanoparticles were cleaned with phosphate-buffered saline (PBS) three times, dried in a desiccator and resuspended to 20 mg/ml (stock solution). Solutions of various concentrations were well dispersed in cell culture medium by sonication for 10 min by using Shumei KQ218 (100 w) Ultrasonic Cleaning equipment (Kunshan Ultrasonic Instruments, Jiangsu, China). All the particles were endotoxin-free, as determined by a quantitative Limulus Amebocyte Lysate (LAL) assay (Charles River, Grand Island, USA) at a detection level of 0.25 % EU/ml.

### Particle-induced murine calvarial osteolysis model

A well-established and widely used model for studying particle-induced osteolysis is the murine calvarial resorption model (Dong et al. 2008; Wang et al. 2013) in which wear particles and other stimuli are placed onto the cranium and their effect on the calvaria is directly assessed. Based on our research purpose and previous studies (Billi and Campbell 2010; Cobb and Schmalzreid 2006; Gill et al. 2012; Polyzois et al. 2012; Tsai et al. 2011; Wang et al. 2013), we introduced the TiAl6V4 (TiNPs) and CoCrMo (CoNPs) nanoparticles (51.7 nm in diameter) to investigate the relationship between particle-induced osteolysis and the ER-stress-mediated inflammatory response within osteolytic tissues in the periprosthetic space. Tg (a classic ER-stress inducer) and 4-PBA (a classic ER-stress blocker) were also employed in the experiments.

Mice weighed 20–25 g at the start of the experiments and techniques described previously were used to construct calvarial models. A total of 70 female C57BL/J6 mice (12–14 weeks old) were randomly divided into seven experimental groups (*n* = 10) evaluated at the Experimental Animal Center of Nanjing University (Nanjing, China): group I, sham surgery controls; group II, TiNPs implantation; group III, TiNPs + 4-PBA co-treatment; group IV, TiNPs + Tg co-intervention; group V, CoNPs implantation; group VI, CoNPs + 4-PBA co-treatment; group VII, CoNPs + Tg co-intervention. As previously described, the mice were anesthetized for the dissection of the cranial periosteum from the calvaria. Then, 40 μl TiNPs/CoNPs suspensions with/without Tg (10^6^ nmol/l) in PBS was spread uniformly under the intact periosteum in the intermediate gap of the cranium. Mice in the 4-PBA groups were injected intraperitoneally with 4-PBA at 300 mg/kg on days 0, 1, 2, 3 and 5 post-surgery.

### Specimen retrieval and histological processing of murine calvarial osteolysis model

The animals were killed in a carbon dioxide chamber at 2 weeks after particle implantation. The calvarial caps were gathered by dissection of the bone from the underlying brain tissue and removal of an elliptical plate of bone bound from the area between the foramina magnum and auditory canals. Then, after being trimmed in half along the midline of the calvaria caps, one half was preserved in pre-cooled PBS for toluidine blue staining, whereas the other was used for HE staining. In addition, osteolytic periosteum tissues were collected and weighed with an analytical balance. One-third was ground in liquid nitrogen and lysed in RIPA lysis buffer in the presence of protein inhibitor cocktails, with the final supernatants being stored at −80 °C for Western blotting and quantitation of Ca^2+^ concentration, ROS level and tartrate-resistant acid phosphatase (TRAP) activity. Another third was homogenized with a high-speed blender in a medium containing appropriate RIPA lysis buffer with protein inhibitor cocktails and the final supernatants were stored at −80°C for ELISA detection. The last third was fixed in 4 % paraformaldehyde for 24 h and the paraffin-embedded tissues were processed for immunohistochemistry. In addition, osteolytic calvarial tissues were also collected for TRAP activity determination.

### Periprosthetic osteolytic bone tissues and interface membrane specimens

Periprosthetic osteolytic bone tissues and interface membrane specimens were obtained at revision operations from patients with periprosthetic osteolysis and aseptic loosening, after infection was ruled out. The unloose specimens came from the acetabulum cup of a patient who underwent a revision operation because of mechanical loosening. Capsule samples from a patient undergoing primary total hip replacement for hip dysplasia served as a control. The clinical data of all patients are shown in Table 1. The interface membrane specimens were collected for TEM, Western blotting, ELISA, immunohistochemistry and determination of Ca^2+^ concentration, ROS level and TRAP activity. In addition, osteolytic bone tissues were also collected for TRAP activity evaluation.

**Table 1:** Clinical data of patients (*F* female, *M* male, *Cup* acetabular cup, *UNL* unloose specimen, *LOO1–6* loose specimens 1–6). UNL and LOO1–6 specimens were collected from the interface of the bone and the artificial femoral head

Case	Gender	Age (years)	Pre-operative diagnosis	Years after implantation	Type of fixation	Specimen collection site
Control	F	17	Hip dysplasia	–	–	Acetabulum
UNL	M	74	Mechanical loosening	10	Cementless	Cup
LOO1	M	47	Aseptic loosening	24	Cementless	Cup
LOO2	M	66	Aseptic loosening	17	Cementless	Cup
LOO3	F	72	Aseptic loosening	3	Cementless	Cup
LOO4	M	67	Aseptic loosening	10	Cementless	Cup
LOO5	M	49	Aseptic loosening	17	Cementless	Cup
LOO6	M	49	Aseptic loosening	17	Cementless	Cup

### Transmission electron microscopy

Osteolytic interface membrane specimens for TEM were fixed in 2.5 % glutaraldehyde in 0.1 mol/l phosphate buffer (pH 7.4) for 1 day and post-fixed in 1 % osmium tetroxide. The fixed specimens were then dehydrated through a graded ethanol series and embedded in Epon 618 (TAAB Laboratories Equipment, Berks., UK). Ultrathin sections were stained with lead citrate and uranyl acetate. Images were captured by a TEM (Philip CM-120) operated under normal atmospheric pressure and temperature.

### Micro-computed tomography scanning

Qualitative and quantitative osteolysis analyses were carried out in mouse calvaria by a high-distinguishability micro-computed tomography (micro-CT) scanner (SkyScan1176; SkyScan, Aartselaar, Belium). An equidistant resolution at 18 μm was used in the scanning protocol, with X-ray energy settings of 45 kV and 550 μA. To reduce negative effects of metal nanoparticles, TiNPs and CoNPs were eliminated before scanning. After reconstruction, the osteolytic region was selected for further analyses. The percentage of total porosity of each calvaria sample was measured as previously reported (Liu et al. 2014).

### Histology and immunohistochemistry

Bone resorption studies were performed on calvaria by using the toluidine blue staining assay. Briefly, the calvaria, whose surfaces were cleanly removed, were placed in 0.25 % trypsin for 15 min and remained overnight in 0.25 M ammonium hydroxide before being stained with 0.25 % toluidine blue for 15 min. Slices were trimmed and mounted on slides with buffering glycerin after vigorous washes and air-drying. A light microscope (Nikon TE2000U, Nikon, Tokyo, Japan) was used to observe the extent of lacunar absorption by calculating the number of absorption pits.

The interface membrane and calvaria tissue sections were stained with HE for standard histological analysis of inflammatory infiltration. Briefly, the paraformaldehyde-fixed and paraffin-embedded sections from the retrieved tissues were sliced at a thickness of 4 μm and stained with HE solution. Four separate sections per sample were assessed in an unbiased manner. The positive cells for targeted protein in immunohistological sections were counted via Image-Pro Plus analysis software 6.0 (Media Cybernetics, Silver Spring, Md., USA).

### Western blotting

The retrieved interface membrane and periosteum tissues were crushed and ground in RIPA lysis buffer (50 mM TRIS, pH 7.4, 150 mM NaCl, 1 % sodium deoxycholate, 1 % Triton X-100, 0.1 % SDS, 1 mM phenylmethane sulfonyl-fluoride) including a cocktail of protease inhibitors; the resulting lysate was cleared by centrifugation at 12,000*g* for 8 min at 4 °C. The supernatants were then collected and determined with a BCA protein assay kit (Biocolor Bioscience and Technology, Shanghai, China) to examine the protein concentration.

The supernatant equivalent of 100 μg protein was electrophoresed on 10 % sodium dodecyl sulfate polyacrylamide gels and transferred onto nitrocellulose membranes. After non-specific blocking with skim milk, the nitrocellulose membranes were incubated, respectively, with rabbit polyclonal antibodies raised against GRP78/Bip, IRE1-α, rabbit polyclonal antibody against NF-κB, c-Fos and D-glyceraldehyde-3-phosphate dehydrogenase (Cell Signaling Technology, Beverly, Mass., USA). Subsequently, anti-rabbit IgG secondary antibodies conjugated to horseradish peroxidase (Santa Cruz, Calif., USA) were applied. After determination of the formula weight of the purified protein by SDS polyacrylamide gel electrophoresis, the band was excised from the gel for protein identification by using a chemiluminescence detection system (Cell Signaling Technology). Band density was analyzed by using ImageJ 1.41 (National Institutes of Health, Bethesda, Md., USA).

### Determination of Ca^2+^ levels

Ca^2+^ concentrations in the interface membrane and periosteum tissues were measured by colorimetric assays by using a Calcium Detection Kit (Abcam, USA). The experimental procedure was in accordance with the manufacturer’s instructions. Briefly, 90 μl color-developing agent and 60 μl calcium assay buffer were blended in each well containing 50 μl sample and mixed gently. The reaction was incubated for 5–10 min at room temperature in the dark. Absorbance was measured at 575 nm and the final results were normalized to total cell protein and presented as millimols per gram protein. Results represent the means ± SD of three experiments performed in duplicate.

### ROS level determination

The intracellular ROS levels in the interface membrane and periosteum tissues were detected by the ROS-specific fluorescent probe 2-,7-dichlorofluorescin diacetate (DCFH-DA; Beyotime, China). All procedures were performed in accordance with the manufacturer’s instructions. Values were obtained at 488 nm excitation and 525 nm emission. The changes in absorbance rate were directly proportional to the ROS levels. The final values were normalized to total cell protein and the results are presented as the means ± SD of three experiments performed in duplicate.

### Tissue TRAP activity and ELISA

TRAP activity in osteolytic bone, calvaria, interface membrane and periosteum tissues was determined by using a tissue TRAP Quantification Kit from Jiancheng Biotech (Nanjing, China). Inflammatory factors (TNF-α, IL-1β, IL-6 and M-CSF) present in the osteolytic interface membrane and periosteum tissues were quantified by using specific ELISA kits. All processes were carried out in accord with the manufacturer’s instructions. Total protein levels were used for normalization.

### Immunohistochemistry

Samples of the interface membrane and periosteum were sectioned into 4-μm-thick slices and mounted on protein-coated slides. The expression of VEGF, OPG and RANKL was analyzed by using immunohistochemical staining of the osteolytic interface membrane and periosteum tissues. After the blocking of endogenous peroxidase activity and immersion of the sections in antigen retrieval buffer, they were incubated with primary antibodies raised against VEGF, OPG and RANKL at 4 °C overnight and then washed three times. The ChemMate EnVision/horeseradish peroxidase solution was mixed, brought to ambient temperature and applied to the sections. Color was developed by adding diaminobenzidine solution, plus counterstaining with hematoxylin. The sections were analyzed to obtain the percentage of cells stained positively for the target protein in various specimens. Eight randomly selected areas were analyzed for each immunostaining. Micrographs were acquired and the total count of positive cells was derived by using Image Pro Plus software (Media Cybernetics, Silver Spring, MD, USA).

### Statistical analysis

Data are presented as means ± standard error of the mean (SEM). Differences in the average of the variables of experimental parameters between groups were evaluated by analysis of variance (ANOVA). Post hoc testing of the differences between each group was implemented by utilizing Duncan’s test when the ANOVA was substantial. A *P*-value <0.05 was deemed to be a significant difference. No adjustment for multiple testing was applied since the statistical analysis was performed in an exploratory way. Statistical analyses were processed with the SPSS v17.0 software package (SPSS, Chicago, Ill., USA).

## Results

### Histopathologic changes of osteolysis mediated by particle-induced ER stress

To address the effect of ER stress on osteolysis and to investigate the relationship between particle-induced osteolysis and the ER-stress-mediated inflammatory response in osteolytic tissues of the periprosthetic space, we produced a particle-induced bone resorption animal model to evaluate directly the impact of ER stress on particle-induced osteolysis. Based on previous studies and our TEM data, we introduced TiAl6V4 (TiNPs) and CoCrMo (CoNPs) nanoparticles, with Tg and 4-PBA being used as an ER-stress inducer and blocker, respectively. In the murine model of periprosthetic osteolysis, no deaths were noted during or after the implantation of TiNPs and CoNPs and mice maintained normal activity throughout the study. At 2 weeks after calvarial bone implantation of TiNPs and CoNPs, mice were killed and the histopathologic changes of particle-induced osteolysis were evaluated by micro-CT scanning and toluidine blue staining.

**Fig. 1:**
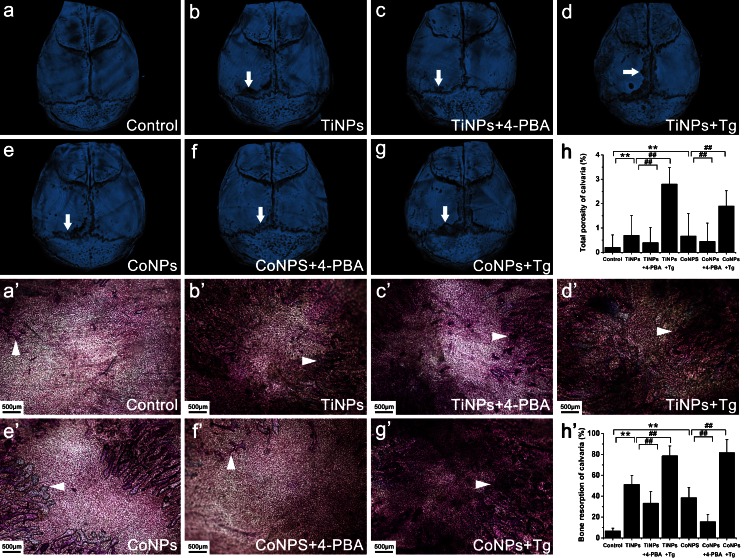
Histological changes of osteolysis mediated by particle-induced endoplasmic reticulum (ER) stress (*TiNPs* TiAl6V4 nanoparticles, *CoNPs* CoCrMo nanoparticles, *Tg* thapsigargin, *4-PBA* 4-phenylbutyrate). **a–h** Representative microtomography images of the calvaria in each group (*arrows* calvaria surface corrosion). **a’–h’** Toluidine blue staining of the calvaria in each group (*arrowheads* bone resorption lacunae). Original magnification: ×40. *Bar* 500 μm. Data represent means ± SEM for each group. *P < 0.05, **P < 0.01 versus control. ^#^ *P* < 0.05, ^##^ *P* < 0.01 versus TiNPs or CoNPs

As expected, implantation of TiNPs and CoNPs induced serious bone resorption, as evidenced by the vast areas of surface corrosion observed on the cranium, compared with the sham surgery controls, which had no implantation of wear particles (Fig. 1a–h). By contrast, the blocking of ER stress with 4-PBA significantly reduced wear-particle-induced bone erosion and destruction. However, the total bone porosity of osteolytic calvaria was exacerbated by co-administration of the ER-stress-inducer Tg (Fig. 1a–h). Histological and histomorphometric analyses after toluidine blue staining further verified the remission of particle-induced bone resorption and destruction by 4-PBA. The degree of lacunar resorption was characterized by calculating the number of resorption pits. As shown in Fig. 1a’–h’, wear-particle implantation resulted in considerable toluidine-blue-positive bone resorption lacunae in the calvarial caps. Consistent with the micro-CT quantitation, histological analysis demonstrated that 4-PBA co-treatment significantly decreased the bone resorption induced by wear particles, whereas the osteolysis areas were markedly increased after TiNPs or the CoNPs and Tg combination (Fig. 1a’–h’). Collectively, these data revealed a key role for ER stress in particle-induced bone erosion and destruction, thus indicating that the blocking of ER stress with a specific inhibitor might be an effective anti-bone resorptive mechanism for the therapy of particle-induced osteolysis.

### Involvement of ER stress in osteolytic periosteum tissues taken from animal models of periprosthetic inflammatory osteolysis

**Fig. 2:**
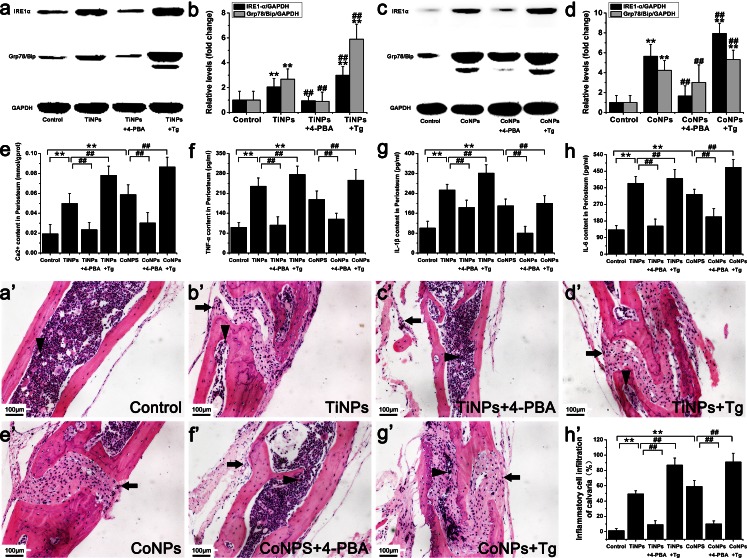
ER stress, pro-inflammatory cytokines and hematoxylin and eosin (HE) staining in animal models of periprosthetic inflammatory osteolysis (PIO). **a-d** Western blots of IRE1α and Grp78/BiP in periosteum tissues performed after animals were treated with various stimuli. The density of the Western blot bands was quantified by using ImageJ software (*GADH* D-glyceraldehyde-3-phosphate dehydrogenase). **e-h** Ca^2+^ content (**e**) and the expression of pro-inflammatory cytokines tumor necrosis factor-α (*TNF-α*, **f**), interleukin-1β (*IL-1β*, **g**) and interleukin-6 (*IL-6*, **h**) in periosteum tissues after animals were treated with various stimuli. **a’–h’** HE staining of the calvaria in each group. Infiltration of inflammatory cells was observed in the calvaria. Inflammatory cells (*arrows*) and osteoclasts (*arrowheads*) were identified according to histomorphologic criteria. Original magnification: ×200. *Bar* 100 μm. Data represent the means ± SEM from three independent experiments. +*P* < 0.05, ++*P* < 0.01 versus control. ^#^ *P* < 0.05, ^##^ *P* < 0.01 versus TiNPs or CoNPs

To investigate whether ER stress was involved in osteolytic periosteum tissues, we monitored the expression of ER-stress-associated molecules IRE1α and GRP78/Bip by Western blot (Fig. 2a–d) and assessed Ca^2+^ leakage by using colorimetric assays (Fig. 2e). As expected, the expression levels of the ER-stress markers (IRE1-α, GRP78/Bip and Ca^2+^) in osteolytic periosteum tissues were all significantly increased as osteolysis progressed following the implantation of TiNPs and CoNPs (Fig. 2a–e). However, ER-stress induction by TiNPs and CoNPs was overtly suppressed by co-treatment with 4-PBA (Fig. 2a–e). In contrast, co-administration of TiNPs or of CoNPs and Tg induced higher ER stress compared with treatments with each product alone (Fig. 2a–e). Thus, these data imply that wear particles are capable of inducing ER stress and Ca^2+^ mobilization in osteolytic periosteum tissues from animal models of periprosthetic inflammatory osteolysis (PIO).

### ER-stress-mediated inflammatory response in osteolytic periosteum tissues from PIO animal models

**Fig. 3:**
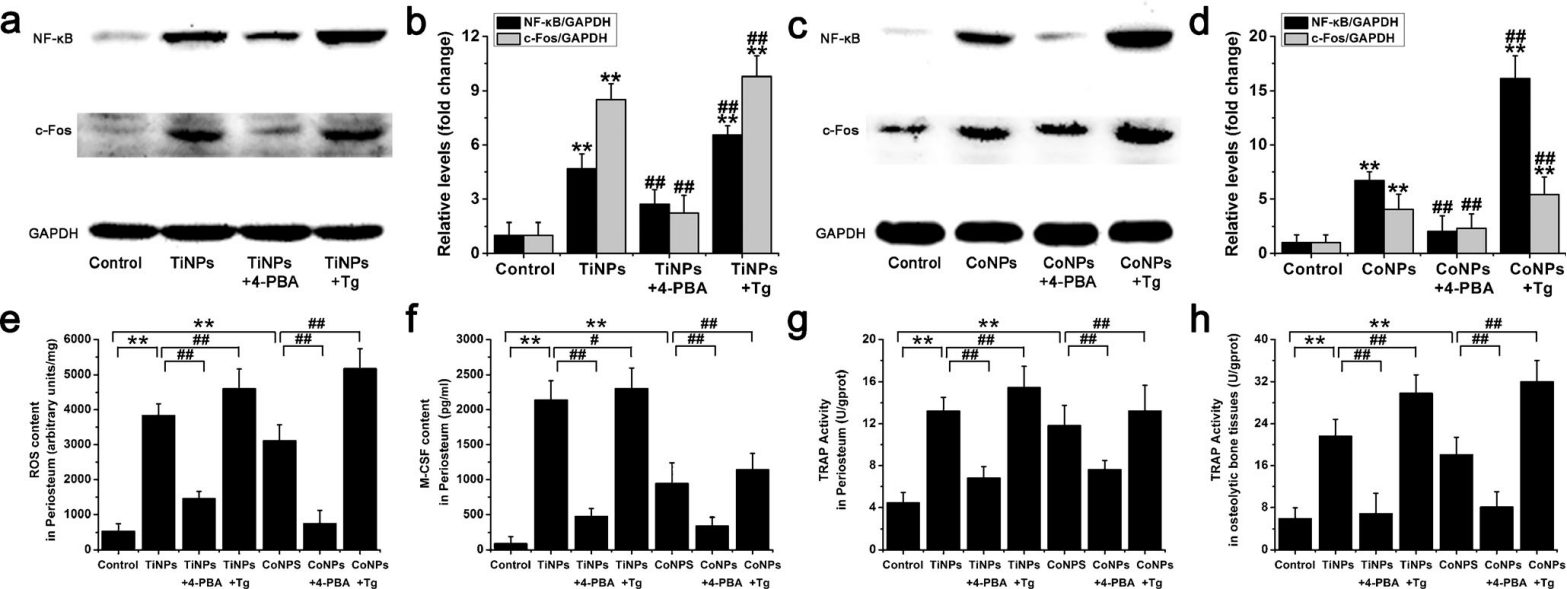
ER-stress-mediated nuclear factor kappa B (*NF-κB*) and c-Fos, reactive oxygen species (*ROS*), macrophage/colony-stimulating factor (*M-CSF*) and tartrate-resistant acid phosphatase (*TRAP*) activity in osteolytic periosteum tissues from PIO animal models. **a-d** Western blots of NF-κB and c-Fos in periosteum tissues performed after animals were treated with various stimuli. The density of the Western blot bands was quantified by using ImageJ software. **e-h** ROS content (**e**), M-CSF content (**f**) and TRAP activity (**g**) in periosteum tissues and TRAP activity in calvaria tissues (**h**) after animals were treated with various stimuli. The data represent the means ± SEM from three independent experiments. +*P* < 0.05, ++*P* < 0.01 versus control.^ #^ *P* < 0.05, ^##^ *P* < 0.01 versus TiNPs or CoNPs

An inspection of ER stress demonstrated many links to the inflammatory pathways, including networks activated by oxidative stress (ROS) and Ca^2+^ and the NF-κB and IRE1α/JNK/c-Fos -AP-1 pathways. As described above, we demonstrated that wear particles induce ER stress via IRE1α, GRP78/Bip and Ca^2+^, exacerbating bone resorption when phagocytosed by macrophages in osteolytic periosteum tissues taken from PIO animal models. Therefore, to investigate whether particle-induced osteolysis and ER stress are relevant to the inflammatory response, we next assessed the expression of pro-inflammatory cytokines (TNF-α, IL-1β and IL-6) by ELISA (Fig. 2f–h), analyzed inflammatory cell infiltration and membrane erosion by using HE staining (Fig. 2a’–h’), examined c-Fos and NF-κB expression levels by Western blot (Fig. 3a–d) and assessed ROS release by using fluorescent probes (Fig. 3e).

As shown in Fig. 2f–h, the implantation of TiNPs and CoNPs induced severe inflammation, as determined by the significantly increased TNF-α, IL-1β and IL-6 levels, compared with negative controls. In contrast, co-treatment with 4-PBA significantly reduced the expression of wear-particle-induced inflammatory cytokines (Fig. 2f–h), However, TNF-α, IL-1β and IL-6 levels in the Tg co-intervention groups showed a completely opposite trend to those obtained for the 4-PBA co-treatment groups (Fig. 2f–h).

Histological analysis of HE-stained osteolytic calvaria further confirmed the reduction of particle-induced inflammation by 4-PBA (Fig. 2a’–h’). Generally, the morphology of control mouse calvaria was smooth and seldom manifested bone erosion and inflammatory cells infiltrates (Fig. 2a’–h’). Bone and inflammatory cells were identified according to histomorphologic criteria. Normally, osteoblast clusters were found in bone matrix juxtaposing the mouse calvaria, whereas inflammatory cells remained on the surface and corroded the calvarial surface (Fig. 2a’–h’). In PIO animal models, implantation of TiNPs and CoNPs induced extensive inflammatory infiltrates, as evidenced by pronounced inflammatory cell infiltration along the eroded bone surface compared with negative controls (Fig. 2a’–h’). In addition, the bone morphology remained intact in controls, whereas increased surface bone erosion was observed in osteolytic calvarial tissues after particle implantation (Fig. 2a’–h’). However, 4-PBA co-treatment significantly reduced particle-induced bone erosion and inflammatory infiltrates (Fig. 2a’–h’). By contrast, chaotic bone morphology, extensive bone erosion and frequent inflammatory cell infiltration were significantly exacerbated by Tg co-intervention (Fig. 2a’–h’). Together with the micro-CT (Fig. 1a–h) and toluidine blue staining (Fig. 1a’–h’) results, these data suggest that wear particles promote osteolysis and inflammatory infiltrates and have an effect highly dependent on ER-stress induction.

Next, we explored whether the expression of c-Fos and NF-κB were induced in the periosteum following the various treatments. As expected, a significant increase in c-Fos and NF-κB expression levels was induced after implantation of TiNPs and CoNPs representing wear particles (Fig. 3a–d). In addition, significant differences in c-Fos and NF-κB expression levels were obtained between the ER-stress inducer (Tg) and ER-stress blocker (4-PBA) groups (Fig. 3a–d). Indeed, c-Fos and NF-κB expression levels were overtly increased with Tg co-administration but were notably inhibited by 4-PBA co-treatment, following TiNPs and CoNPs implantation (Fig. 3a–d). The above results revealed that c-Fos and NF-κB expressions were mediated by ER stress and that the inhibition of ER stress could limit c-Fos and NF-κB production in osteolytic periosteum tissues.

Furthermore, we examined the ROS activities in osteolytic periosteum tissues and found that they increased as osteolysis progressed following the implantation of TiNPs and CoNPs (Fig. 3e). However, ROS activity levels after implantation of TiNPs and CoNPs were markedly inhibited by co-administration of 4-PBA (Fig. 3e). By contrast, the effects were significantly aggravated by co-intervention with Tg (Fig. 3e).

Thus, the results obtained above demonstrated that the inflammatory response in osteolytic periosteum tissues was induced by wear particles, a predominantly ER-stress-mediated effect. Moreover, the inhibition of ER stress effectively reduced the inflammatory response in osteolytic periosteum tissues from PIO animal models.

### Expression of ER-stress-mediated osteoclastogenic molecules in osteolytic periosteum tissues from PIO animal models

Previous studies have revealed that osteoclasts differentiation is dependent on M-CSF and RANKL, whose expression is up-regulated during osteoclast differentiation. VEGF, the most potent angiogenic growth factor, plays an important role in particle-induced inflammation and osteoclast differentiation. Simultaneously, inflammatory and osteoclastogenic pathways also have crosslinks in their associated signaling cascades, including the IRE1α/JNK/c-Fos-AP-1 and NF-κB pathways. Given our findings that particle-induced GRP78/Bip and IRE1α (Fig. 2a–d), c-Fos and NF-κb (Fig. 3a–d) expression levels were obviously mediated by ER stress, we hypothesized that ER stress might also be responsible for the expression of osteoclastogenic enzymes and molecules, such as M-CSF, TRAP, VEGF, OPG and RANKL. To test this hypothesis, tissue levels of M-CSF, TRAP, VEGF, OPG and RANKL were assessed.

As shown in Fig. 3f, a low expression of M-CSF was observed in normal periosteum tissues. Implantation of TiNPs and CoNPs, however, resulted in large M-CSF amounts. In addition, M-CSF content was highest in the Tg co-intervention group and was drastically reduced when animals were co-treated with 4-PBA (Fig. 3f).

**Fig. 4:**
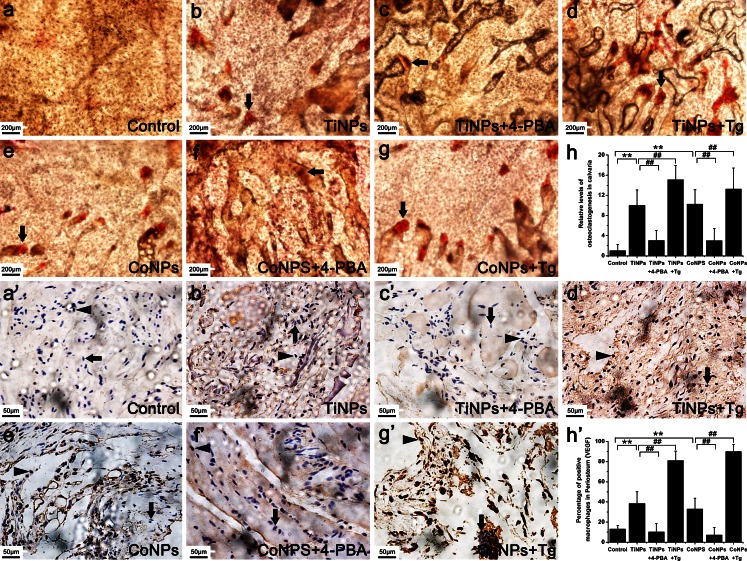
Particles increased osteoclast numbers and VEGF immunostaining. **a–h** Histochemical staining for TRAP in calvaria from animals treated with various stimuli. Osteoclasts (*arrowheads*) were identified according to histomorphologic criteria. Original magnification: ×100. *Bar* 200 μm. **a’–h’** Immunohistochemistry for VEGF in periosteum tissues from animals treated with various stimuli. Positively stained macrophages (*arrowheads*) and normal macrophages (*arrows*) were identified according to histomorphologic criteria. Original magnification: ×400. *Bar* 50 μm. Data represent the means ± SEM from three independent experiments. *P < 0.05, **P < 0.01 versus control. ^#^ *P* < 0.05, ^##^ *P* < 0.01 versus TiNPs or CoNPs

To investigate whether the effects of osteolysis caused by ER stress in vivo were relevant to the increased osteoclast number, TRAP quantification kits were used to determine the capabilities of osteoclastogenesis in osteolytic periosteum (Fig. 3g) and bone (Fig. 3h) tissues. In addition, we also compared the number of osteoclasts among the various treatment groups by histochemical staining for TRAP, a cytochemical marker for osteoclasts (Fig. 4a–h). We found that TRAP activity and osteoclast numbers following TiNPs and CoNPs implantation were higher than those of negative controls. Consistent with our M-CSF data (Fig. 3f), the effects were suppressed by co-treatment with 4-PBA and exacerbated by co-administration of Tg (Figs. 3g, h, 4a–h), suggesting that the differentiated osteoclasts were significantly increased by exposure to wear particles and that ER stress is involved in particle-induced osteoclastogenesis in PIO animal models.

**Fig. 5:**
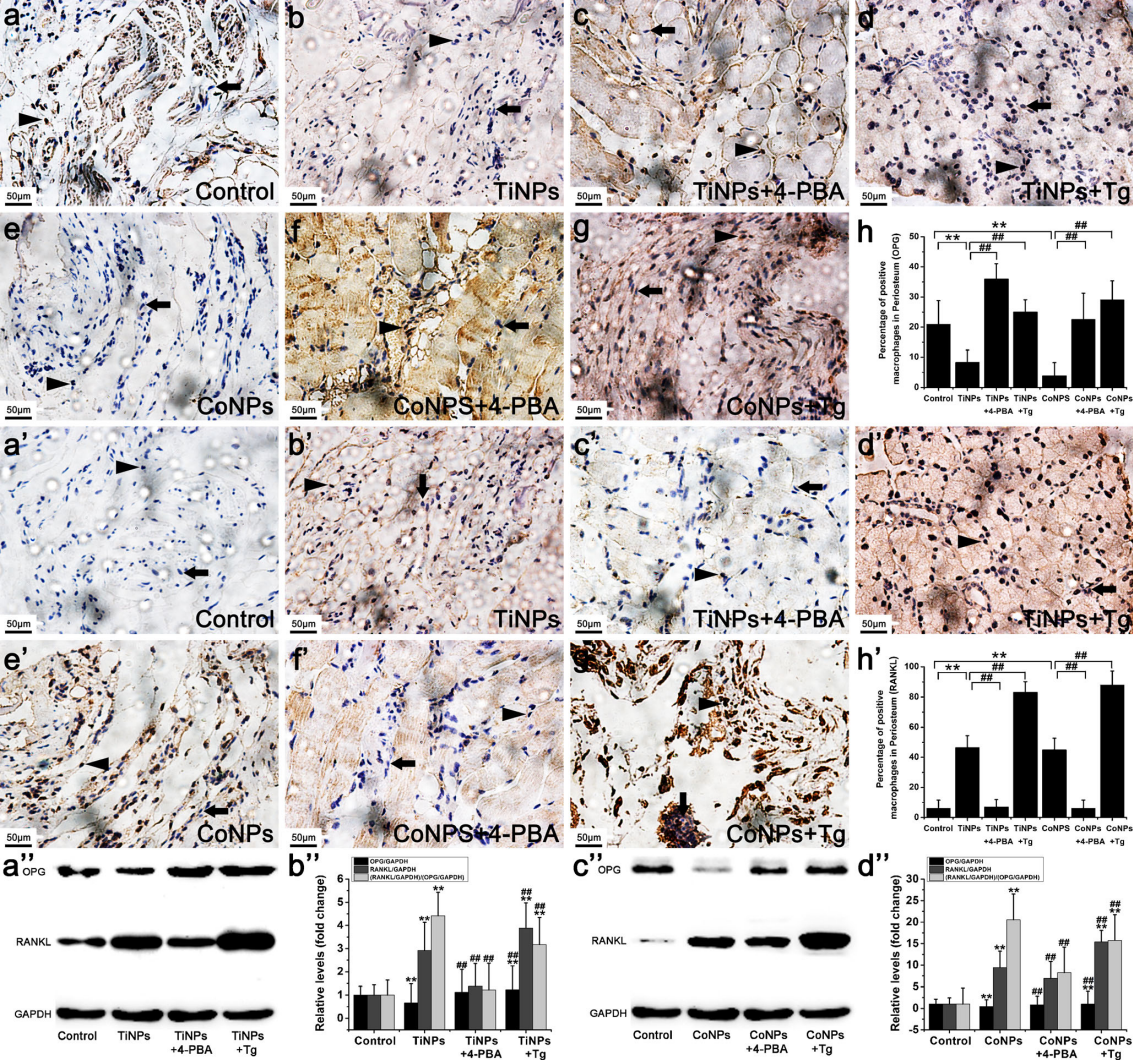
Osteoprotegerin (*OPG*) and receptor activator of nuclear kaapa B (*RANKL*) expression levels in PIO animal models. **a–h**, **a’–h’** Immunohistochemistry of OPG (**a–h**) and RANKL (**a’–h’**) in periosteum tissues from animals treated with various stimuli. Positively stained macrophages (*arrowheads*) and normal macrophages (*arrows*) were identified according to histomorphologic criteria. Original magnification: ×400. *Bars* 50 μm. **a’’–d’’** Western blots of OPG and RANKL in periosteum tissues performed after animals were treated with various stimuli. The density of the Western blot bands was quantified by using ImageJ software. Data represent the means ± SEM from three independent experiments. *P < 0.05, **P < 0.01 versus control.^ #^ *P* < 0.05, ^##^ *P* < 0.01 versus TiNPs or CoNPs

We next searched for cells positive for VEGF (Fig. 4a’–h’), OPG (Fig. 5a–h) and RANKL (Fig. 5a’–h’) in retrieved osteolytic periosteum tissues by means of immunohistochemistry. The numbers of macrophages positive for VEGF (Fig. 4a’–h’) and RANKL (Fig. 5a’–h’) significantly increased after TiNPs and CoNPs implantation, whereas their frequency in normal tissues was lower compared with osteolytic periosteum tissues. To investigate further whether particle-induced VEGF and RANKL expression resulted from ER stress, we examined the effect of Tg and 4-PBA on particle-induced VEGF and RANKL expression levels. As expected, cells positive for VEGF and RANKL were most intensive in the TiNPs or CoNPs plus Tg co-intervention groups and the effects were reduced starkly in the 4-PBA co-treatment group (Figs. 4a’–h’, 5a’–h’). In addition, the trend of OPG changes was different from those of VEGF and RANKL. As shown in Fig. 5a–h, only a few positive cells were observed in the osteolytic periosteum tissues following the implantation of TiNPs and CoNPs compared with normal tissues. However, OPG expression was highly expressed in the TiNPs or the CoNPs plus 4-PBA co-treatment groups (Fig. 5a–h).

Given that the RANKL/OPG ratio is suitable for estimating osteolysis, the quantitative analysis of RANKL and OPG was examined by Western blot. The RANKL and OPG expression levels obtained by Western blot (Fig. 5a’’–d’’) corroborated the immunohistochemistry data (Figs. 5a–h, a’–h’). In addition, this ratio was obviously increased in the TiNPs and CoNPs groups, compared with the PBS control group (Fig. 5a’’–d’’). The RANKL/OPG ratio in the Tg co-intervention group was only slightly increased compared with the PBS control group but was slightly decreased compared with that in the osteolytic periosteum tissues implanted with TiNPs and CoNPs (Fig. 5a’’–d’’). In addition, the RANKL/OPG ratio after 4-PBA co-administration was overtly decreased compared with that of the TiNPs and CoNPs groups (Fig. 5a’’–d’’).

Collectively, these findings suggested that the production of osteoclastogenic molecules in osteolytic periosteum tissues from PIO animals was mediated by ER stress and that the blocking of ER stress with 4-PBA could impair osteoclastogenesis.

### Pre-operative X-ray examination and tissue ultrastructural analysis

**Fig. 6:**
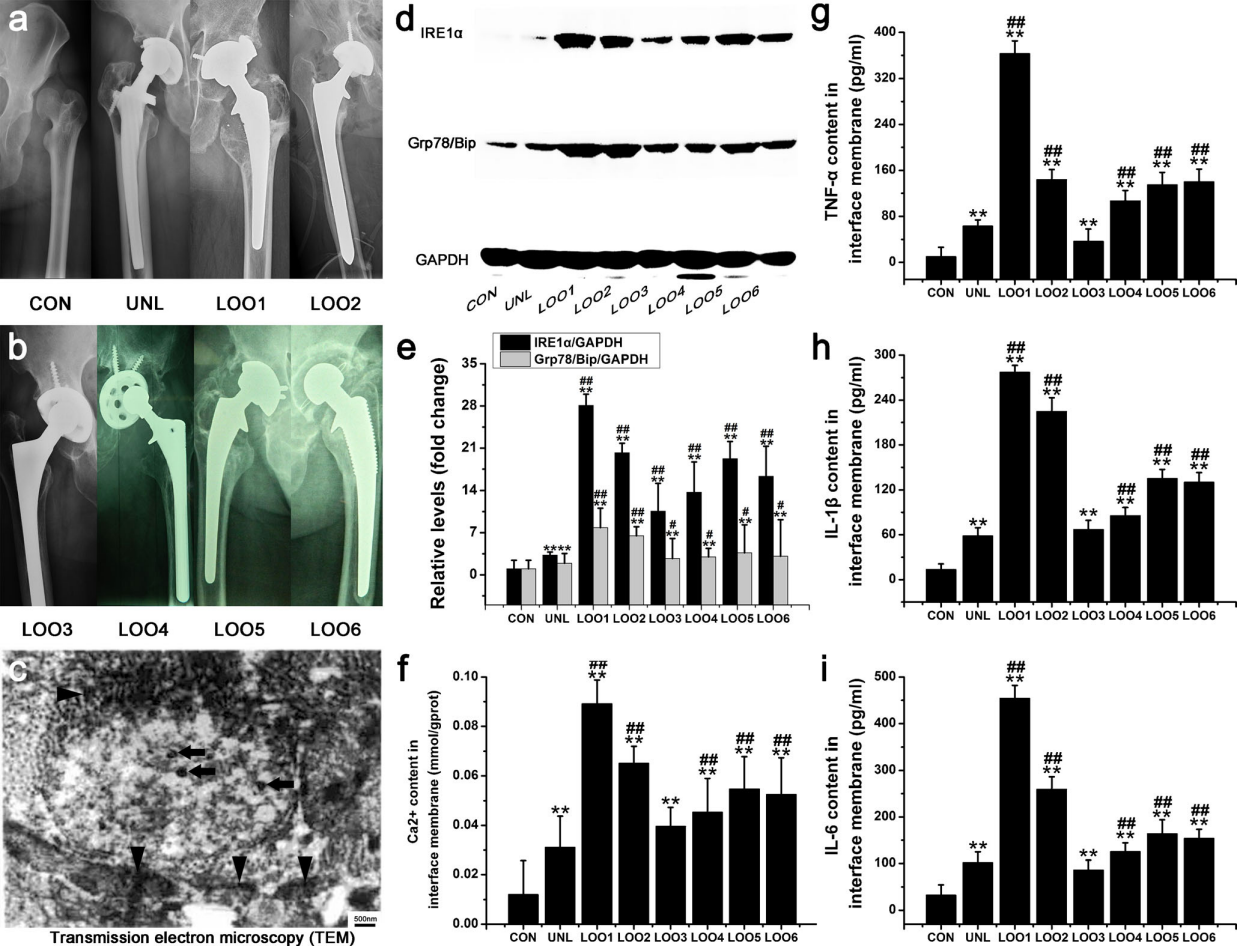
Pre-operative X-ray examination, tissue ultrastructure analysis, ER stress and pro-inflammatory cytokines in interface membrane tissues of patients with aseptic loosening (*CON* control, *UNL* unloose specimen, *LOO1–6* loose specimens 1–6). **a**, **b** Pre-operative radiographs of patients. **c** Ultrastructure of osteolytic interface membrane. *Bar* 500 nm. Metal wear particles (*arrowheads*) and cellular structures (*arrows*) were identified according to histomorphologic criteria. **d**, **e** IRE1α and GRP78/Bip expression levels were increased in patients with aseptic loosening. The density of the Western blot bands was quantified by using ImageJ software. **f-i** Ca^2+^ content (**f**) and the expression of pro-inflammatory cytokines TNF-α (**g**), IL-1β (**h**) and IL-6 (**i**) were increased in patients with aseptic loosening. Data represent the means ± SEM from three independent experiments.*P < 0.05, **P < 0.01 versus CON. *#P* < 0.05, *##P* < 0.01 versus UNL

Pre-operative X-rays of patients are shown in Fig. 6a, b. Patients in our study all showed definite osteolysis and loosening signs in X-rays. In our study, we found that the extent of bone resorption evaluated by intra-operative observation was in agreement with pre-operative radiographs and the implantation time of the revision patient. In addition, the ultrastructure of the osteolytic interface membrane tissues was revealed by TEM. As shown in Fig. 6c, a large number of black metal wear particles were found in osteolytic interface membrane tissues. They were mainly of a rounded outward appearance, with smooth edges and a diffuse distribution and accumulated into agglomerations. Analysis of TEM images revealed that the particles were in the nanometer range, with the majority showing a size of 30–60 nm. In addition, the size distribution of these wear particles within the osteolytic interface membrane tissues in our study was in accordance with previous reports.

### Involvement of ER stress in clinical osteolytic interface membrane tissues

To investigate whether ER stress is involved in the osteolytic interface membrane, we examined IRE1α, GRP78/Bip and Ca^2+^ levels in clinical specimens. As expected, IRE1-α and GRP78/Bip expression levels in the osteolytic interface membrane were all significantly increased compared with normal hip dysplasia tissues and with tissues after mechanical loosening (Fig. 6d, e). Ca^2+^ content was also significantly increased as osteolysis progressed (Fig. 6f). In addition, the data also suggested a correlation between ER-stress intensity and the clinical severity of osteolysis. Among these patients, the most severe bone resorption, according to pre-operative radiographs and intra-operative observation, was obtained in patient LOO1 (Fig. 6a, b). ER-stress intensity was also the highest in this patient (Fig. 6d–f). However, IRE1-α, GRP78/Bip and Ca^2+^ levels showed slight changes in the patient with mechanical loosening relative to the control (Fig. 6d–f). According to the integrated analysis, the ER-stress pathway was involved in particle-induced osteolytic interface membrane tissues during osteolysis, whereas the clinical severity of osteolysis and ER-stress intensity were inter-related.

### Inflammatory response is aggravated by exacerbation of osteolysis progression

**Fig. 7:**
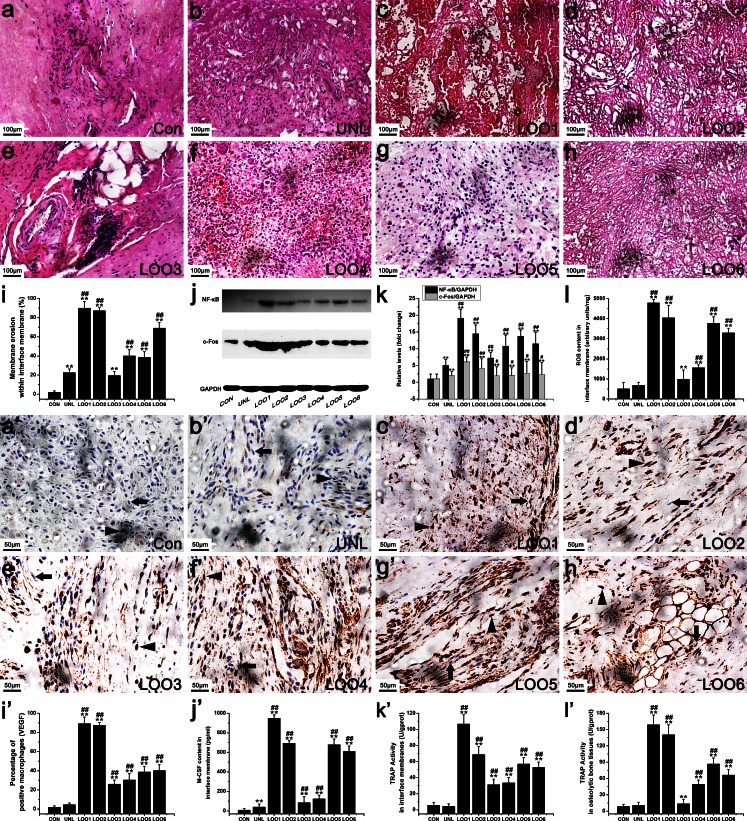
Up-regulation of membrane erosion, NF-κB and c-Fos expression levels, ROS content, VEGF immunostaining, M-CSF content and TRAP activity in patients with aseptic loosening (*CON* control, *UNL* unloose specimen, *LOO1–6* loose specimens 1–6). **a–i** Membrane erosion surface was increased in patients with aseptic loosening. Original magnification: ×200. *Bars* 100 μm. **j**, **k** NF-κB and c-Fos expression levels were increased in patients with aseptic loosening. The density of the Western blot bands was quantified by using ImageJ software. **l** ROS content was increased in patients with aseptic loosening. **a’–i’** Photomicrographs of immunostaining in interface membrane tissues of patients with aseptic loosening showing that several macrophages were positively labeled for VEGF. Positively stained macrophages (*arrowheads*) and normal macrophages (*arrows*) were identified according to histomorphologic criteria. Original magnification: ×400. *Bars* 50 μm. **j’–l’** M-CSF content (**j’**) and TRAP activity (**k’**) in interface membrane and TRAP activity in osteolytic bone tissues (**l’**) were increased in patients with aseptic loosening. Data represent the means ± SEM from three independent experiments. *P < 0.05, **P < 0.01 versus CON.^ #^ *P* < 0.05, ^##^ *P* < 0.01 versus UNL

Our results described above demonstrated that wear particles in the osteolytic interface membrane are capable of inducing ER stress via IRE1α, GRP78/Bip and Ca^2+^ and of exacerbating bone resorption when phagocytosed by macrophages in the periprosthetic space. Therefore, to investigate whether the particle-induced osteolysis and ER stress described above are relevant to the inflammatory response, we next assessed the pro-inflammatory cytokines (TNF-α, IL-1β and IL-6) expression (Fig. 6g–i), analyzed membrane erosion (Fig. 7a–i), examined c-Fos and NF-κB expression levels (Fig. 7j, k) and assessed ROS release (Fig. 7l) in our patients.

Tissue levels of TNF-α, IL-1β and IL-6 were quantified by ELISA and the results are shown in Fig. 6g–i. As expected, low TNF-α, IL-1β and IL-6 expression levels were obtained in normal hip dysplasia tissues and in tissues after mechanical loosening. Osteolytic interface membrane tissues, however, expressed robust amounts of TNF-α, IL-1β and IL-6 (Fig. 6g–i). Histological analysis of an HE-stained interface membrane was used to assess membrane erosion (Fig. 7a–i). The membrane remained smooth and intact in normal hip dysplasia tissues and in tissues after mechanical loosening, whereas an increased membrane erosion surface and decreased membrane thickness were observed in osteolytic interface membrane tissues (Fig. 7a–i). We then found that the c-Fos and NF-κB expression levels in osteolytic interface membrane tissues were significantly increased compared with normal hip dysplasia tissues and with tissues after mechanical loosening (Fig. 7j, k). This indicated that the c-Fos and NF-κB expression levels increased with the severity of osteolysis. Additionally, ROS levels gradually increased with the exacerbation of osteolysis (Fig. 7l).

More importantly, we also found that the pro-inflammatory cytokine contents (Fig. 6g–i), membrane erosion surface (Fig. 7a–i), c-Fos and NF-κB expression levels (Fig. 7j, k) and ROS concentration (Fig. 7l) shown above were in agreement with each other and followed the same trend as ER stress (Fig. 6d–f) and osteolysis symptoms (Fig. 6a, b). These findings suggested that the particle-induced inflammatory response in the osteolytic interface membrane significantly increased with osteolysis progression, especially in the moderate and severe stages of osteolysis compared with normal tissues and with tissues after mechanical loosening. In addition, the clinical severity of osteolysis and ER-stress intensity might have a certain relationship with the content and form of the inflammatory response.

### Up-regulation of osteoclastogenic molecules with exacerbation of osteolysis

To investigate the potential involvement of osteoclastogenic molecule activation in the osteolytic interface membrane, the expression levels of the osteoclastogenic factors M-CSF, VEGF, OPG and RANKL and the amounts of the osteoclast maturation enzyme TRAP were quantified.

**Fig. 8:**
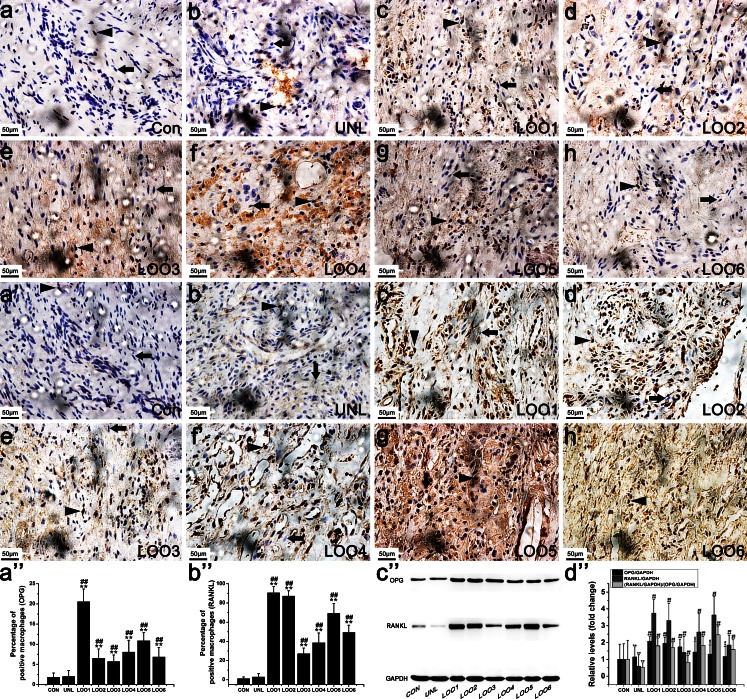
Up-regulation of OPG and RANKL expression levels in interface membrane tissues of patients with aseptic loosening (*CON* control, *UNL* unloose specimen, *LOO1–6* loose specimens 1–6). **a–h**, **a’’**, **a’–h’**, **b’** Photomicrographs of immunostaining in interface membrane tissues of patients with aseptic loosening showing that several macrophages were positively labeled for OPG (**a–h**) and RANKL (**a’–h’**) and quantification of OPG (**a’’**) and RANKL (**b’’**) expression levels. Positively stained macrophages (*arrowheads*) and normal macrophages (*arrows*) were identified according to histomorphological criteria. Original magnification: ×400. *Bars* 50 μm. **c’**, **d’** OPG and RANKL expression levels were increased in patients with aseptic loosening. The density of the Western blot bands was quantified by using ImageJ software. Data represent the means ± SEM from three independent experiments. *P < 0.05, **P < 0.01 versus CON. ^#^ *P* < 0.05, ^##^ *P* < 0.01 versus UNL

As depicted in Fig. 7f’–i’, significantly increased VEGF immunostaining was observed with increasing osteolysis severity and was consistent with the M-CSF levels (Fig. 7j’) and TRAP activity data (Fig. 7k’, l’). The expression levels of OPG and RANKL were determined by immunohistochemical staining (Fig. 8a–h, a’’) and Western blot (Fig. 8a’–h’, b’’). In normal tissues, as expected, only low expression of OPG and RANKL was detected. In the osteolysis stage, however, macrophages in the osteolytic interface membrane showed robust expression of RANKL accompanied by the slight expression of OPG. Thus, our results described above suggest that the osteoclastogenic molecules are significantly up-regulated with the progression of osteolysis symptoms.

The wear particles were therefore capable of inducing osteolysis and ER stress and were associated with the inflammatory response and the expression of osteoclastogenic molecules. The blocking of ER stress with 4-PBA dramatically decreased particle-induced ER stress and osteolysis symptoms. Simultaneously, this ER-stress blocker also lessened inflammatory cell infiltration, diminished the capability of osteoclastogenesis and reduced the inflammatory response through the inhibition of specific biomarkers (IRE1α, GRP78/Bip, c-Fos, NF-κB, ROS and Ca^2+^) of the ER-stress-induced inflammatory signaling pathways.

## Discussion

Aseptic loosening caused by osteolysis is a severe long-term side-effect after joint replacement and can negatively affect the longevity and prolonged efficacy of the prosthesis (Beck et al. 2012; Park et al. 2005; Purdue et al. 2006). Therefore, an understanding of the characteristics and loosening mechanisms of joint replacement, specifically with respect to preventative action, is essential for the purpose of reducing the prospective revision burden. Unfortunately, non-operative therapy is mostly unavailable to resolve this process in the earlier stages of periprosthetic osteolysis; only the radiological proof of aseptic loosening necessitates revision surgery (Geng et al. 2010a; Goodman et al. 2005; Mao et al. 2012). However, with the improved comprehension of the molecular and cellular biological processes involved in particle-induced osteolysis, pharmacological interventions targeting inflammation and osteoclastogenesis have emerged as promising approaches to recognize this detrimental process and to ameliorate the deterioration of artificial prosthesis at the molecular level (Chen et al. 2012; Goodman et al. 2005; Ren et al. 2004, 2006a, 2010; Yang et al. 2012). Since the current prevention and treatment methods of osteolysis are barely adequate, considerable scientific investigation is required with regard to alternative treatment options for the prevention and inhibition of periprosthetic osteolysis.

The continuous enduring inflammatory osteolysis that follows total joint replacement has its origin in the repeated inflammatory-cell-mediated phagocytosis of wear particles at the bone/prosthesis interface and is characterized by the constitution of the fibrous interface membrane around the loosening prosthesis (Ren et al. 2006b; Wang et al. 2004). Analysis of the fibrous interface membrane revealed the domination of macrophages, which represent 60–80 % of the whole cell population (Gallo et al. 2014). Wear particles generated from prostheses are phagocytosed by tissue-resident macrophages, resulting in their activation, sustained stimulation and phagocytic effects and eventually leading to inflammatory response and osteoclast differentiation. Accumulating evidence indicates that particle-induced continuous inflammation and osteoclast differentiation are two decisive factors in the pathogenesis of osteolysis and aseptic loosening and that enhanced osteoclastogenesis caused by the inflammatory response is the final pathway of bone resorption (Chen et al. 2012; Goodman et al. 2005; Ren et al. 2004, 2006a, 2010; Yang et al. 2012). Since osteoclasts derive from the macrophages lineage, various studies (Pandey et al. 1996; Quinn et al. 1992; Sabokbar et al. 1997) have indicated that tissue-resident macrophages and some macrophages recruited to the fibrous interface membrane around loosening prostheses are capable of differentiating into osteoclasts. Given that the activation of macrophage is not limited to tissues surrounding the loosening prosthesis but extends to the circulating monocytes in the mononuclear phagocyte system, the chances are that macrophages represent both the “origin” and “amplification” of the pathogenetic chain reaction in periprosthetic osteolysis. Therefore, the interaction between macrophages and wear particles in the interface membrane around failed implants might be alternatively considered a contributing detrimental factor in osteolysis or a preventive element of osteolysis.

**Fig. 9:**
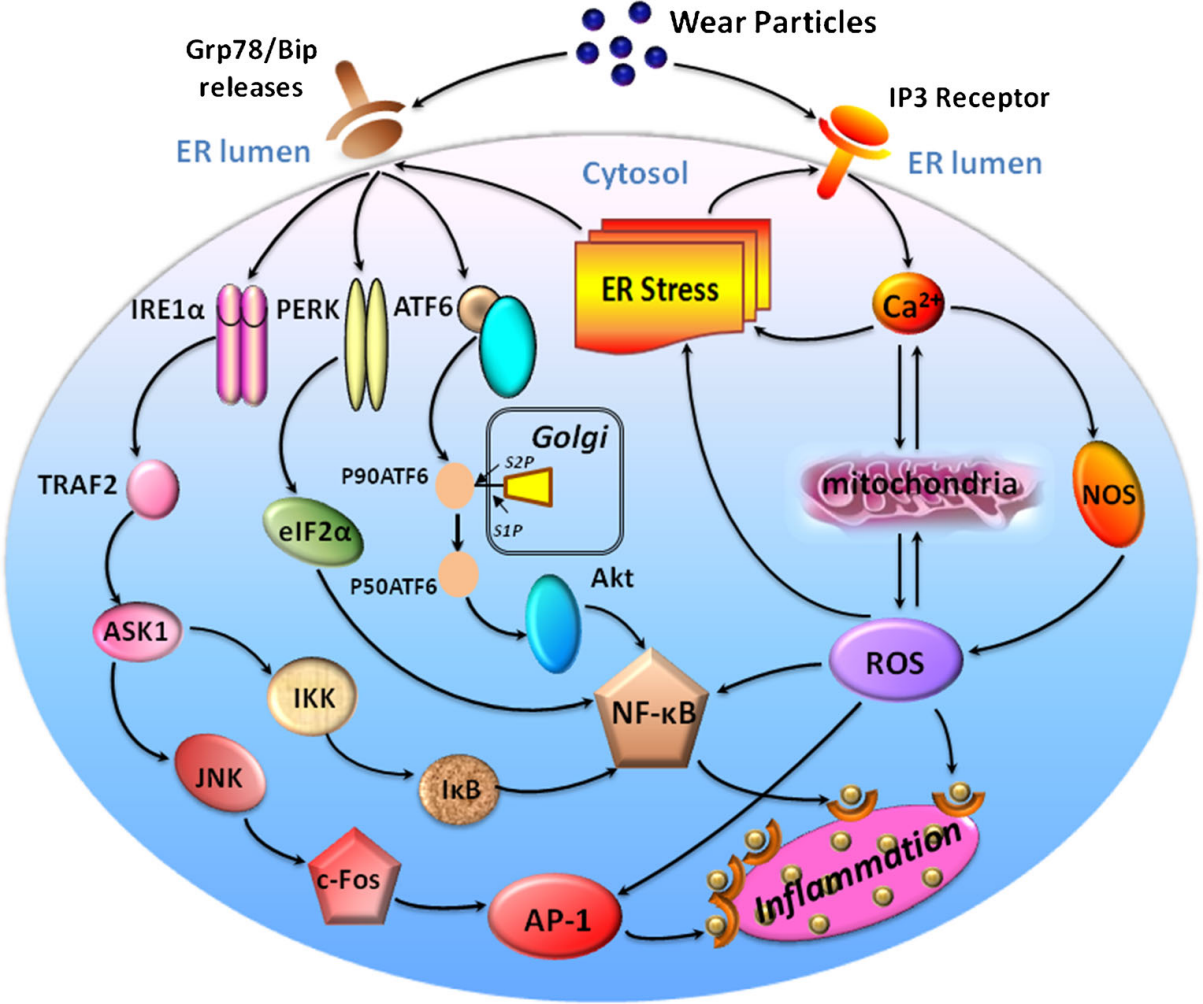
ER-stress-mediated inflammatory signaling pathways proposed in particle-induced periprosthesis osteolysis. Wear particles phagocytosed by macrophages might induce ER stress and several potential avenues might link ER stress and inflammatory signaling. Normally, the ER chaperone Grp78/BiP binds the N-termini of ER-stress sensors IRE1α, PERK and ATF6, preventing their activation. In response to particle-induced ER stress, Ca^2+^ is released from ER and Grp78/BiP preferentially binds to unfolded or misfolded proteins, thus driving the equilibrium of Grp78/BiP binding away from IRE1α, PERK and ATF6. Upregulation of these genes triggers ER stress and thereby activates their downstream mediators of inflammation and leads to the release of NF-κB, AP-1 and ROS. These molecules move to the nucleus and switch on the expression of a variety of genes involved in inflammation, such as TNF-α, IL-1β and IL-6, leading to inflammatory responses

The local inflammatory response, whereby macrophages represent the predominant cell-type, is recognized as a critical mechanism leading to osteoclast differentiation and subsequent osteolysis (Abu-Amer et al. 2007; Goodman et al. 2013; Purdue et al. 2006). Many cellular signaling transduction pathways and cytokines have been demonstrated to be related to the process of the inflammatory response but the exact mechanism is still not comprehended. Recently, a batch of signal pathways that target cellular stress have been distinguished (Hotamisligil 2010; McGuckin et al. 2010; Xu et al. 2005; Zhang and Kaufman 2008). Together, they are known as ER-stress pathways and studies of ER stress have widened the comprehension of the mechanisms by which inflammation originates. Many studies suggest that ER-stress signaling pathways and inflammation are interconnected through the activation of the IRE1α/JNK/c-Fos-AP1 and NF-κB pathways, ROS production and the release of Ca^2+^ from the ER (Hotamisligil 2010; McGuckin et al. 2010; Xu et al. 2005; Zhang and Kaufman 2008) (Fig. 9). Notably, these signaling pathways and mechanisms also play a principal role in particle-induced inflammatory osteolysis. For instance, the Ti-particle-induced release of TNF-α, IL-1β and IL-6 in macrophages occurs via the activation of the JNK/AP-1 and NF-κB pathways (Ren et al. 2004). Cell culture and in vivo animal models (Kong et al. 2013; Liu et al. 2014; Nakashima et al. 1999; Ren et al. 2004; Schwarz et al. 2000; Zhai et al. 2014) have confirmed that wear particles bring about the progression of inflammatory osteolysis by the activation of principal pathways in macrophages, principally the NF-κB and JNK/c-Fos-AP-1 signaling cascades. As previously reported (Hotamisligil 2010; Zhang and Kaufman 2008), IRE1α is probably critical for the integration of ER stress with inflammatory signaling via the activation of both JNK/c-Fos-AP-1and NF-κB in response to ER stress. In addition, the IRE1α, PERK and ATF6 subfield of ER stress has been linked to NF-κB signaling, suggesting that specific triggers of inflammation act as signals via various branches of the ER-stress pathway. Consistent with these findings, other studies (Kong et al. 2013; Liu et al. 2014; Nakashima et al. 1999; Ren et al. 2004; Schwarz et al. 2000; Zhai et al. 2014) have demonstrated that inhibitory approaches targeting the NF-κB and AP-1 signal transduction pathways ameliorate particle-induced osteoclastogenesis, inflammation and osteolysis in vitro and in vivo.

Inflammatory and osteoclastogenic pathways also have crosslinks in their associated signaling cascades, including the JNK/c-Fos-AP-1 and NF-κB pathways (Hotamisligil 2010; Zhang and Kaufman 2008). Meanwhile, other studies (Cheng et al. 2010; Lin et al. 2014; Liu et al. 2014; Yamanaka et al. 2011, 2013; Zhai et al. 2014) have shown that wear particles induce osteoclastogenesis in part by the stimulation of RANK and M-CSF and the activation of the NF-κB and JNK pathways. These reports also indicate the feasibility of the pharmaceutical and molecular blockade of the NF-κB and JNK pathways to inhibit particle-induced osteoclastogenesis. Previously (Liu et al. 2014; Yamanaka et al. 2013), the suppression of JNK signaling has been shown to be able to inhibit particle-induced osteoclastogenesis and osteolysis and the wear-particle induction of mature osteoclast formation from RANKL-primed osteoclast precursor cells is suppressed by an NF-κB depressor (Zhai et al. 2014). In agreement, we demonstrated that the ER-stress inhibitor 4-PBA ameliorates particle-induced inflammatory osteolysis and osteoclastogenesis by inhibiting the NF-κB and JNK/c-Fos-AP-1 activation pathways and by suppressing the ER production of ROS and Ca^2+^.

Because of the function of macrophages as scavengers, the protracted stimulation and repeated phagocytosis of wear particles will continuously activate the cellular machinery related to intracellular degradation, such as lysosomes, peroxisomes and proteolytic enzymes (Rae 1986). Thus, one of the direct results of this process is the biogenesis and accumulation of ROS and Ca^2+^ (Shanbhag et al. 1998; Tucci et al. 2000), which could crucially affect polypeptide folding and chaperone function, initiating ER stress. However, enduring ER stress might also lead to oxidative stress and Ca^2+^ imbalance, inciting the accumulation of intracellular ROS and Ca^2+^ leakage from the ER; this might trigger the activation of NF-κB and c-Fos-AP-1 signaling and initiate an inflammatory response (Cullinan and Diehl 2006; Gorlach et al. 2006; Hotamisligil 2010; Zhang and Kaufman 2008). Previous studies (Nakajima and Kitamura 2013; Raghunathan et al. 2013) have provided evidence that ROS generation is induced by various wear particles in macrophages. Localized ROS production has also been implicated in eliciting cytokine over-expression at the prosthesis/bone interface (Nakajima and Kitamura 2013). However, the molecular mechanisms underlying ROS-induced ER stress and inflammation have not been completely elucidated. Recent research (Zhang and Kaufman 2008) has demonstrated that ROS induces the depletion of the Ca^2+^ store in ER via the suppression of Ca^2+^-ATPase, the production and accumulation of oxidatively-modified proteins and the functional perturbation of ER chaperones. These molecular events trigger the unfolded protein response, thereby causing ER stress and leading to NF-κB and c-Fos-AP-1 regulation. Moreover, experiments (Pahl et al. 1996) with anti-oxidants and Ca^2+^ chelators have revealed that Ca^2+^ and oxidative stress signals bring about the activation of NF-κB in reaction against ER stress. Consequently, ER-related NF-κB and c-Fos-AP-1 activation might result from oxidative stress and/or from the ER-stress-mediated release of Ca^2+^ into the cytosol. Additionally, the Ca^2+^ leakage mediated by ER stress is concentrated in the mitochondrial matrix and results in the depolarization of the inner mitochondrial membrane, the disruption of electron transport and the increase in ROS production. ROS accumulation in mitochondrial can further promote Ca^2+^ release from ER by sensitizing ER Ca^2+^-liberating channels and resulting in ER stress. It is through such a forward cycle that the continuous development of Ca^2+^ leakage, ROS generation and ER stress together activate Ca^2+^-dependent protein kinases and the IRE1α/JNK/c-Fos-AP1 and NF-κB pathways, exacerbating inflammatory responses (Fig. 8).

In the present clinical experiments, we found that the levels of the specific biomarkers of the ER-stress-mediated inflammatory signaling pathways (IRE1α, GRP78/Bip, c-Fos, NF-κB, ROS and Ca^2+^), inflammatory factors (TNF-α, IL-1β and IL-6), and osteoclastogenic molecules (VEGF, OPG, RANKL and M-CSF) within the osteolytic interface membrane all increase with the deterioration attributable to osteolysis progression. These data indicate that the inflammatory signaling pathway is involved in particle-induced osteolysis; in addition, the ER-stress pathway, inflammatory signaling pathway, osteoclastogenic reaction and clinical severity of osteolysis are linked to one another.

Having established that wear particles indeed accelerate the inflammatory response and ER stress in clinical situations, we also explored its inductive effects under pathological osteolysis conditions in a PIO animal model. We produced a wear-particle-induced osteolysis animal model to validate the necessary relationship between ER stress and particle-induced inflammatory signaling pathways and directly to evaluate the effects of ER stress on localized particle-induced osteolysis. Based on our study aim, on previous studies (Billi and Campbell 2010; Cobb and Schmalzreid 2006; Gill et al. 2012; Polyzois et al. 2012; Tsai et al. 2011; Wang et al. 2013) and on our TEM results, we introduced TiNPs and CoNPs, with a mean particle diameter of 51.7 nm, into our model. We demonstrated that the blocking of ER stress with 4-PBA dramatically decreases particle-induced ER-stress intensity and osteolysis symptoms in vivo. Interestingly, this ER-stress blocker also lessens inflammatory cell infiltration, decreases the release of inflammatory factors (TNF-α, IL-1β and IL-6), diminishes the capability of osteoclastogenesis by suppressing the expression of osteoclastogenic cytokines (VEGF, RANKL and M-CSF) and reduces the inflammatory response through inhibition of the specific biomarkers of the ER-stress-mediated inflammatory signaling pathways (IRE1α, GRP78/Bip, c-Fos, NF-κB, ROS and Ca^2+^). However, the effects discussed above are significantly aggravated when the ER-stress inducer (Tg) is used in combination with wear particles.

In addition, we found high-level OPG expression in the normal group, whereas wear particles TiNPs and CoNPs can reduce this reaction. RANKL is slightly elevated in the sham surgery controls but is markedly increased in the TiNPs and CoNPs groups, indicating that OPG does not confer protection against bone resorption induced by wear particles. However, RANKL or OPG cannot be used independently to evaluate osteoclast differentiation, as the widespread coordination between them is indispensable to manage bone erosion. The ability or inability of 4-PBA to induce RANKL expression might be important in determining whether OPG is able to protect against particle-induced osteoclastogenesis. As expected, OPG expression is raised in the TiNPs or the CoNPs plus 4-PBA co-treatment groups and yet protects against particle-induced bone resorption; meanwhile, 4-PBA co-treatment leads to reduced RANKL levels compared with those exposed to particles with implantation. The decreased RANKL levels together with the moderately increased OPG amounts might be sufficient to shift the balance toward osteoclastogenesis. This property of 4-PBA might reduce the influence of RANKL and diminish the excretion of pro-inflammatory and pro-osteoclastogenic cytokines. These findings are consistent with previous studies (Baumann et al. 2004; Granchi et al. 2004; Wang et al. 2010) demonstrating the reduction of OPG secretion after particle stimulation but differ from observations describing an obvious secretion of OPG (Bylski et al. 2009; Mao et al. 2012; Ren et al. 2009). Given the differences between the wear nanoparticles used here and the microparticles of previous studies, we assume, like other researchers (Baumann et al. 2004; Bylski et al. 2009; Granchi et al. 2004; Mao et al. 2012; Ren et al. 2009; Wang et al. 2010), that the different compositions and physical characteristics of wear particles affect the secretion of cytokines related to inflammatory osteolysis.

Great progress has been made in comprehending the signaling pathways that integrate ER stress, apoptosis, inflammation, osteoclastogenesis and the physiological significances of these associations. However, we still face many problems and should continue to carry out intensive research to improve our understanding of the interrelationship between them. Recently, several studies (Clohisy et al. 2004; Geng et al. 2010a, 2010b; Jin et al. 2011; Kong et al. 2013; Landgraeber et al. 2009, 2014; Lee et al. 2013; Liu et al. 2009, 2014; Mao et al. 2012; Wang et al. 2013; Zhai et al. 2014) have concentrated on exploiting efficacious medical treatment for osteolysis by regulating the apoptotic, inflammatory and osteoclastogenic responses. However, modulation of the interlinkage between these basic biological responses for medical purposes, without giving rise to severe untoward effects, is a considerable challenge. Since apoptosis, inflammation and osteoclastogenesis interact with each other, any independent action of one of these signaling pathways is impossible with respect to taking charge of the pathogenesis of osteolysis. Given their complexity, the most effective way of implementing changes would be to seek to re-establish biological homeostasis by regulating the incorporated functional outcomes rather than the targeting of single pathways.

Recent studies (Boyce et al. 2005; Ozcan et al. 2006; Wang et al. 2013) have suggested that the preservation or restoration of ER function might be therapeutic in chronic metabolic diseases. In our study, we demonstrated that ER stress stimulates the induction of inflammatory reactions and subsequent osteolysis as a result of particles in the osteolytic periosteum tissues taken from PIO animal models and clinical specimens of prosthesis loosening. The blocking of ER stress with 4-PBA dramatically suppresses osteoclast differentiation, decreases inflammatory cytokine expression and reduces osteolysis severity. Taken together, our findings provide a proof of principle in a preclinical setting that the ER can be chemically targeted to broaden its potential function and such a strategy might open up attractive possibilities for preventative action and the treatment of patients with artificial joint replacement who are at high risk of early inflammatory osteolysis. Future studies should address the physiological significance of the various ER-stress pathways in mediating the inflammatory and osteoclastogenic response.

In the present study, we found that wear particles generated at the implant interface can be distributed throughout the periprosthetic milieu, can occupy adjacent tissues and can accelerate ER-stress-mediated inflammatory and osteoclastogenic responses, eventually leading to periprosthetic osteolysis. The blocking of ER stress with 4-PBA dramatically decreases particle-induced ER-stress intensity and osteolysis symptoms. Meanwhile, this ER-stress blocker also lessens the infiltration of inflammatory cells, diminishes the capability of osteoclastogenesis and reduces the inflammatory response through the inhibition of the specific biomarkers (IRE1α, GRP78/Bip, c-Fos, NF-κB, ROS and Ca^2+^) of the ER-stress-mediated inflammatory signaling pathways. The above results confirm that ER stress plays a significant role in particle-induced inflammatory osteolysis and osteoclastogenic reactions. We present a diagram to show the inflammatory signaling pathways mediated by ER stress within the osteolytic interface membrane in particle-induced periprosthetic osteolysis (Fig. 9). A fascinating prospect is now opening up regarding pharmacological targeting by using drugs for macrophages in the interface membrane and related repair signaling pathways in the ER in order to alleviate problems in or even allow the regeneration of the damaged periprosthetic tissues.
